# Toward an Effective Long-Term Strategy for Preventing Motor Vehicle Crashes and Injuries

**DOI:** 10.3390/ijerph110808123

**Published:** 2014-08-11

**Authors:** Anthony R. Mawson, E. Kenneth Walley

**Affiliations:** 1 School of Health Sciences, College of Public Service, Jackson State University, 350 West Woodrow Wilson Avenue, Room 229, Jackson, MS 39213, USA; 2 2174 Henry Hill Drive, Suite 2, Jackson, MS 39204, USA; E-Mail: ekwalley@gmail.com

**Keywords:** injury, prevention, safety, roads, technology, robotics

## Abstract

Casualties due to motor vehicle crashes (MVCs) include some 40,000 deaths each year in the United States and one million deaths worldwide. One strategy that has been recommended for improving automobile safety is to lower speed limits and enforce them with speed cameras. However, motor vehicles can be hazardous even at low speeds whereas properly protected human beings can survive high-speed crashes without injury. Emphasis on changing driver behavior as the focus for road safety improvements has been largely unsuccessful; moreover, drivers today are increasingly distracted by secondary tasks such as cell phone use and texting. Indeed, the true limiting factor in vehicular safety is the capacity of human beings to sense and process information and to make rapid decisions. Given that dramatic reductions in injuries and deaths from MVCs have occurred over the past century due to improvements in safety technology, despite increases in the number of vehicles on the road and miles driven per vehicle, we propose that an effective long-term strategy for reducing MVC-related injury would be continued technological innovation in vehicle design, aimed at progressively removing the driver from routine operational decision-making. Once this is achieved, high rates of speed could be achieved on open highways, with minimal risk of crashes and injury to occupants and pedestrians.

## 1. Introduction

Despite major reductions in vehicular injuries and deaths over the past century, motor vehicle crashes (MVCs) remain a significant public health problem in the United States and an increasing problem worldwide. There are over 6 million crashes every year in the U.S., resulting in 2.9 million injuries and 30,000 to 40,000 deaths. Rear-end collisions occur every 8 seconds in the U.S., accounting for a third of all crashes (about 2 million) and causing many head and neck injuries [1]. MVCs were the world’s ninth leading cause of death in 1990, accounting for about one million deaths, but are projected to become the fifth leading cause of death by 2020 [2]. In the U.S., drivers aged 15–20 years comprise 8%–9% of the population and 6%–7% of all licensed drivers, and are involved in 19% of MVC-related fatalities annually [3]. Crashes involving drivers in this age group cost the U.S. economy an estimated $42.3 billion each year [4]. These statistics and projections point to an urgent need to develop an effective long-term strategy for reducing MVC-related casualties worldwide. 

One strategy that has been recommended is to lower speed limits both on major highways and on urban/suburban roads and to enforce them with speed cameras. It was suggested that lower speed limits would reduce the risk of injury and have other salutary effects. On the other hand, motor vehicles can be hazardous even at low speeds, and lowering speed limits on open highways would be detrimental for commerce. Can safe vehicular travel be achieved without lowering speed limits? It is suggested that the true limiting factor in vehicular safety is not speed itself but the capacity of human beings to sense and process information and make rapid decisions. In this paper, we propose that MVC-related injury can be reduced while simultaneously allowing for high rates of speed by progressively freeing the driver from routine vehicular operations. This strategy represents a logical extension of the many successful “passive” methods of injury prevention that have been introduced over the past century as a result of technological advances, leading to marked declines in crash-related deaths, even while the number of vehicles and miles-driven-per-vehicle have increased. Technological innovation to date has mostly led to improvements in crashworthiness and the reduction of roadway hazards. Future developments can be expected to lead to the complete automation of routine vehicle operations and traffic flows, thereby effectively eliminating most MVCs and allowing for safe and greatly increased rates of speed than are possible at present.

## 2. Traffic Fatalities: A Brief History

The first recorded MVC-related death of a pedestrian was in 1896 and the first driver died in a crash in 1898. Since then, MVC-related injuries and deaths have risen dramatically. It was only when changes in driver behavior occurred in 2008, due to rising gasoline prices, that annual traffic deaths in the U.S. came close to being reduced to under 40,000 for the first time since 1961 [5]. The total number of traffic deaths worldwide continues to rise as a function of increasing population as well as increasing numbers of vehicles and miles driven per vehicle. Over six times as many people were driving at the end of the 20th century as in 1925, and the number of vehicles increased 11-fold to approximately 215 million. The number of miles traveled in motor vehicles was also 10 times higher than in the mid-1920s. The International Red Cross has described the last 100 years as the “Century of road death” [6].

**Figure 1:**
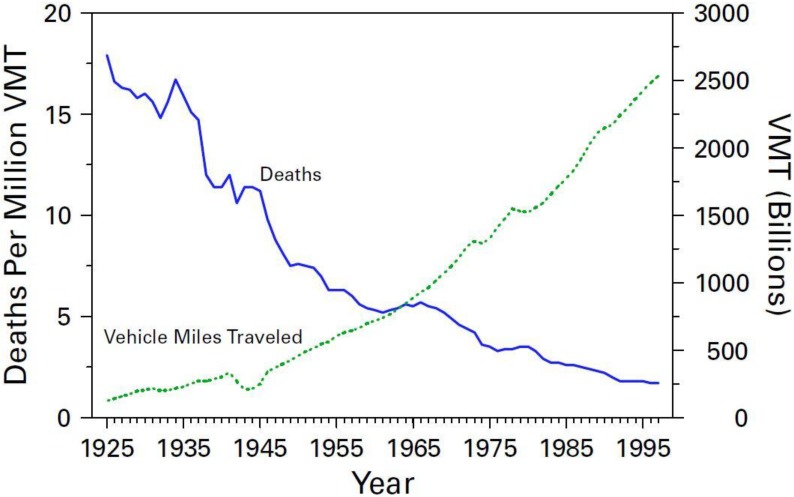
Motor-vehicle-related deaths per million vehicle miles traveled (VMT) and annual VMT, by year—United States, 1925–1997.

Source: MMWR [7].

On the other hand, MVC fatality rates per vehicle registered and distance travelled per vehicle have steadily declined over the past century, with much lower fatality rates in the 1990s than in the 1930s. In fact, MVC death rates have declined by 94% over the past century, from 150 deaths per billion km travel in 1921 to 9.4 deaths per billion km travel in 2002, representing one of the greatest achievements of public health in the 20th century (Figure 1) [7,8]. As noted by Evans [9], if the 1921 rate had applied in 2002, the number of U.S. traffic fatalities in 2002 would have exceeded half a million, *i.e*., 10 times higher than the number of such deaths in 2005 (n = 45,520) [7].

Many environmental, behavioral, and medical factors have contributed to declining MVC death rates, including technological changes and engineering efforts that improved the safety of vehicles and highways as well as successful efforts to change personal behavior [7,10,11,12,13]. Among them were the following:

- Improvements in the crashworthiness of vehicles, such as rubberized bumpers, impact-reducing body materials, improved braking systems, center high-mounted brake lights, seat belts, child safety seats, air bags, and padded dashboards;
- Improved highway surfacing, grading and general engineering, including human factors-based design of intersections and hazardous stretches of highway;
- Improved lighting, warning and directional signage;
- The removal of trees and other fixed objects close to the road; and
- Behavioral changes such as decreased drinking and driving.

The period from 1973–1975 was associated with a slight decrease in crash fatalities, coinciding with the introduction of 55 mph speed limits. 

## 3. The Case for Lowering Speed Limits 

Most MVC-related injuries result from exchanges of mechanical energy that exceed the tolerance threshold of the human body [14]. From this perspective the factors that contribute to road injuries can be categorized similarly to other public health problems, *i.e*., factors related to the host (the human beings affected by the injury), the agent (the sources of the mechanical energy transferred to the host), and the environment (e.g., the physical characteristics of the roadway) [15,16]. Consistent with these precepts, speed limits would be expected to reduce energy exchanges in MVCs and thereby lower the risk of severe injury and death. Friedman *et al*. [17] tested this hypothesis by assessing changes in death and injury rates resulting from fatal crashes during the period 1995–2005 on U.S. urban interstate and non-interstate roads, following the 1995 repeal of the federally-imposed 55 mph speed limit. Raising the speed limits on all types of roads resulted in a 3.2% increase in road fatalities, especially on rural interstates (9.1%) and to a lesser extent of urban interstate highways (4.0%), and involved an estimated 12,545 deaths and 36,583 injuries in fatal crashes over the 10-year period. On this basis, Friedman *et al*. recommended reintroducing the 55 mph speed limit and enforcing it with speed cameras. While acknowledging that other protective measures introduced between 1995 and 2005 may have partially offset the adverse effect of the rise in speed limits (e.g., increased seat belt use, new laws requiring increased child restraint use, mandatory dual front airbag laws, enforcement of driving-while-intoxicated laws, and other factors), they suggested that reintroducing a 55 mph speed limit would save years of productive life and reduce the cost of MVCs. Other benefits would include reduced gasoline consumption and reduced air pollutant emissions. 

Friedman *et al*. [17] noted that in countries where speed limit camera networks have been introduced and enforced on urban roads (e.g., the United Kingdom, Australia and France), immediate and sustained 40%–50% reductions in deaths occurred following the posting of speed limits on urban roads. With each fatality costing about $1 million and the cost of each injured person at $1.1 million, according to Department of Transportation estimates in 2002 [17], the expected 10-year cumulative cost of repealing the 55 mph speed limit was $12 billion for fatalities alone. Hence, it was proposed that lower legal speed limits and improved enforcement using speed cameras would reduce travel speeds and hence fatalities and associated costs.

Additional support for lowering speed limits comes from a study in London, England, on the impact of introducing 20 mph (32 km/h) traffic speed zones on road crashes, injuries and fatalities [18]. This was an observational study using geographically coded police data on road casualties during the period 1986–2006. Changes in numbers of road injuries within road segments were studied over time using conditional fixed-effects Poisson models. The effect of introducing 20 mph speed zones on casualties within those zones and in adjacent areas was estimated, after adjusting for the underlying downward trend in traffic casualties. Grundy *et al*. [18] found that 20 mph zones were associated with a 41.9% (95% CI: 36.0% to 47.8%) reduction in road casualties. The percentage reduction was larger in children (48.5%) and larger for deaths and serious injuries than for minor injuries. There was also no evidence of “casualty migration” to areas adjacent to 20 mph zones, where casualties also fell by 8% on average. The authors estimated that 20 mph zones would prevent 27 deaths a year and 200 casualties overall, among which 57 would be pedestrians. Based on these findings, Grundy *et al*. [18] recommended that 20 mph zones should be introduced in major cities in Britain and elsewhere. 

In an accompanying editorial, Ameratunga [19] noted that speeding has increased over time; that pedestrians and cyclists are at much greater risk of injury than occupants of cars; and excessive speed is the single most important contributor to road fatalities worldwide. [20] Increased average speeds result in greater risks of crashes and more severe injuries. For instance, a 5% increase in average speed is estimated to result in a 10% increase in crashes that cause injury and a 20% increase in fatal crashes [21]. These risks are greater for pedestrians, who have an 80% risk of being killed at a collision speed of 50 km/h (31 mph), whereas most car occupants will survive if appropriately restrained in well-designed vehicles [20]. Of further note, for every 100 injured motorists admitted to hospitals in England, at least 68 pedestrians and cyclists are injured [22]. A review of interventions aimed at changing driver behavior and reducing traffic speeds suggested that 20 mph zone signs alone would reduce average speeds only minimally, whereas their use with other traffic calming measures such as road humps could reduce average speeds by 10 mph [23].

As noted by Ameratunga [19], the evidence of Grundy *et al*. [18] supports so-called “safe system” approaches to speed management, which allow for human error but attempt to reduce injury risks by limiting and managing speed. Methods include setting and enforcing speed limits, engineering interventions such as road humps and roundabouts, and public education. Reducing speed limits to 30 mph in built-up areas with a mix of vulnerable road users and motor vehicles is a key attribute of a “safe system” and an important step toward achieving this vision. Yet only 29% of 174 countries set speed limits of 31 mph (50 km/h) or lower on urban roads.

## 4. Is Lowering and Enforcing Legal Speed Limits the Most Effective Strategy for Reducing Road Casualties? 

Speed limits are clearly essential in built-up areas and on many rural roads, but are they needed on interstate highways, which were the focus of the study by Friedman *et al*. [17]? The nationwide 55 mph speed limit was first introduced in the U.S. in 1973 to conserve fuel and reduce dependence on foreign oil but had the unanticipated effect of reducing traffic deaths and injuries. Reducing speed limits to 55 mph would therefore be expected to reduce crash-related casualties, as suggested by Friedman *et al*., [17] just as it did when the 55 mph speed limit was first imposed [24]. As noted, faster average speeds result in greatly increased risks of crashes and the severity of crash-related injuries, since the kinetic energy in a crash is the square of the speed rather than being a linear function [25,26].

In fact, however, of the 34,017 fatal motor vehicle crashes in 2008, the highest percentage (29%) occurred at 55 mph, whereas speeds of 65 mph and over accounted for 20% of the total fatalities, comparable to those of 40–45 mph, which accounted for 19%. Most deaths (77%) occurred at speeds of 55 mph and under [27]. A 55 mph speed limit would also have accompanying costs, e.g., related to surveillance and enforcement, prolonged travel time, and a potential overall negative impact on the economy. Given that crash-related deaths and severe injuries can occur at low speeds, why not set the speed limit on open roads even lower? Setting the speed limit at 40 mph would reduce the risk of casualties even more, although public acceptance of such a measure would be unlikely. In the 1960s, before the era of seatbelts and airbags, it was already known that 45% of fatal crashes occur when cars are moving at 40 mph or less, speeds which do not necessarily result in fatal injury if cars are properly designed; moreover, well protected stunt drivers have experienced repeated frontal car crashes at 35 mph without sustaining any injury [28].

Given that lower speeds do not necessarily guarantee safety and crashes at higher speeds do not necessarily result in injury if the occupants are properly tethered and protected, a speed limit of 55 mph can be considered arbitrary. The 55 mph limit also raises questions about the inherent capacity of the average driver to negotiate roads and vehicular traffic at speeds much in excess of that limit. Indeed, we suggest that no matter how safe and crashworthy the motor vehicle may be, the true limiting factor in motor vehicle safety is not speed itself but the limited capacity of human beings, isolated from each other in their own vehicles and with inherent visual restrictions, to sense and process information and make rapid decisions that have positive outcomes.

Human operators of vehicles are assumed to be skilled, alert and focused on the task at hand, but this ideal is seldom met in practice. Aside from physical and cognitive impairments that impose legal restrictions on driving, impairment can take the form of risk-taking and intoxication due to alcohol or drugs; in fact, over 40% of MV-associated deaths are alcohol-related [29]. Other behavioral risk factors include fatigue, rage, anxiety and depression. With regard to fatigue and the risk of falling asleep at the wheel, a recent interview survey of 1000 drivers by the British road safety charity Brake [30] found that 31% of drivers on average (45% of male drivers and 22% of female drivers) admitted to “head-nodding” while driving; that is, so-called micro-sleeps, lasting from 2–30 sec. Actual “falling asleep” at the wheel was reported by 14% of male drivers and 2% of female drivers. Almost half (49%) of the drivers also reported driving after having had less than five hours’ sleep [31]. 

Drivers are also increasingly distracted by secondary tasks such as the use of cell phones and texting. A report on distracted driving fatalities in the U.S. in 2009 included the following list of associated distractions: other occupants in the car, eating, drinking, smoking, adjusting radio, adjusting environmental control, reaching for object in car, and cell phone use. In that year, when there were 30,797 fatal MVCs involving 45,230 drivers, 33.808 people were killed. “Distraction” was reported for 11% (5,084) of the drivers involved in fatal crashes [32]. In addition in 2009 about 2.2 million people were injured in MVCs, of which 448,000 (20% of the injured) involved driver distraction. Drivers under age 20 were the most likely to be distracted. Among drivers under age 20 involved in fatal crashes, 16% were distracted while driving [32]. Driver inattention is estimated to contribute to 20% to 50% of all police-reported crashes, and driver distraction, a sub-category of inattention, from 8% to 13% of all MVCs [33]. A recent study on the performance of secondary tasks and the risk of crashes and near-crashes used a variety of objective methods to record driver behavior [34]. Among experienced drivers the risk of a crash or near-crash increased significantly with cell phone dialing but not with talking on a cell phone. Among novice drivers, such risks increased significantly with cell phone dialing, sending or receiving text messages, reaching for an object other than a cell phone, looking at a roadside object, and eating. More risky than cell phone use is text messaging, which is becoming increasingly common. A study using video cameras installed in the cabs of commercial trucks and buses during a 3-month period found that texting increased the risk of collision as much as 23-fold [35].

Even the most experienced drivers are only partially aware of potential hazards at any given time while operating a vehicle under ideal conditions. Young drivers in particular are at greater risk due to inexperience and are more prone to participate in high-risk behaviors [36]. For instance, teenagers often drive at night with other teenagers, which substantially increases their risk of a crash [37]. When all of these factors are combined with inadequate driving skills, a low rate of safety belt use, excessive speeds, alcohol consumption and distraction due to teenage passengers, electronic gadgetry and texting, crash injury rates accelerate rapidly [38].

## 5. Safety Technology: An Alternative Long-Term Strategy 

Proposals to introduce speed limits to save lives have many historical precedents, starting in the late 19th Century when modern civilization was still in the age of the horse. At that time, motor cars (“horseless carriages”) were widely seen as excessively noisy and a public danger. In Britain, the Red Flag Act required a man to walk with a red flag in front of the vehicle to give warning and keep its speed to a safe 4 mph. This law was repealed in 1896, after which pedestrians were responsible for their own safety. In 1929, a Pedestrians’ Association was formed to protest the deaths of 6000 people a year who were being killed on the roads, half of them pedestrians [39].

The history of ideas regarding motor vehicle safety began with the notion that driver error was the primary cause and that driver education was the key to prevention [12]. Yet long experience has shown that driver education has little impact on MVCs and death rates. A systematic review of 28 studies indicated that the most effective interventions are health promotion campaigns aimed at preventing childhood injuries, e.g., through increasing bicycle and motorcycle helmet use, promoting children's car seat and seatbelt use, traffic calming, and specific legislation against drunk driving. Driver improvement and education courses, on the other hand, are associated with increased crash involvement and violations [13]. 

Recognizing that excessive exchanges of mechanical energy during collisions are responsible for most MVC-related injuries, William Haddon and his associates focused on changes in vehicle and roadway design as offering new opportunities for prevention [15,40]. This profound shift in perspective—from driver behavior to the energy exchanges that actually cause injury—suggested that MVCs and injuries could be largely eliminated by redesigning vehicles and highways. This stimulated technological innovations that improved the crashworthiness of vehicles and led to major reductions in MVC-related injuries and deaths. For instance, seat belts and airbags restrain occupants from becoming missiles, and energy-absorbing vehicle interiors reduce the risk of severe injury when vehicles crash into objects [41]. Safety measures that protect people automatically (“passive prevention”), such as airbags, are much more effective than those that require conscious thought and action (“active prevention”), such as seatbelts. This has meant that injuries can be largely prevented by improving the “packaging” of vehicle occupants and by other safety measures on roads and highways that are independent of driver behavior [42]. Considering that vehicles have also been capable of speeds in excess of 80 mph for most of the past 100 years, the continuous long-term decline in MVC death rates can be attributed primarily to improvements in technology, particularly changes that enhanced the crashworthiness of vehicles.

Proposals to lower speed limits as a means of reducing MVCs and injuries, although consistent with Haddon’s precepts, assume a human operator. By current standards, the fully licensed driver is sufficiently skilled to operate a motor vehicle, in part because MVCs and injuries are so rarely experienced on a personal level compared to the population of drivers in the aggregate that their occasional occurrence is considered an acceptable risk of vehicle operation. Currently, the safety of vehicular travel depends on the collective ability and capacity of drivers to remain error-free and to avoid lapses of attention due to falling asleep, illness, preoccupation with life crises or emergencies, drugs or alcohol, or other distractions. However, now that drivers are increasingly equipped for instant and continuous connectivity to others, distractibility due to electronic devices has become a fixture of modern life and is unlikely to be affected by regulation. In 2009, American adults sent 29.7 text messages on average per day, rising to 39.1 per day in 2010. Mobile users aged 18 to 24 sent 109.5 messages per day on average, or 3200 messages per month [43].

This trend suggests that drivers as a whole are becoming so distracted by electronic communications and other secondary tasks that they pose an increasing threat to themselves and others. It may be necessary to assume, therefore, that distraction is a new way of life and to consider the policy of adapting vehicles to human behavior rather than requiring humans to change their behavior. Instead of lowered speed limits or other active measures of prevention, growing evidence suggests that a more effective long-term strategy for reducing MVC-related injury would be continued technological innovation in vehicle design and the progressive computerization of driver operations to the point where human beings—the main cause of most collisions—can be excluded from the equation of vehicle safety. The long-term goal would therefore be the creation of driverless but supremely safe robotic vehicles. Once this is achieved, high rates of speed could become routine on open highways, with minimal risk of crashes and injury to occupants and pedestrians. 

This scenario would not necessarily depend on present or future methods of vehicle propulsion. Developments in solar technology may obviate the need for fossil fuels and dependence on imported oil, re-energizing molecules from air or water to make liquid fuels. The process involves solar concentrators generating temperatures of >1500 °C that, when combined with catalysts in chemical reactors, split water or carbon dioxide to produce hydrogen gas or carbon monoxide. These chemicals can then be used as energy-rich feed stocks to make liquid fuels such as gasoline. The technology has been shown to work on a small-scale but is not yet commercially feasible [44].

Operator failure (the “human factor”) remains the single greatest obstacle to safety in vehicular travel. Hence, an initial goal for preventing MVCs could be the universal use of braking systems that can override the driver when necessary to prevent frontal collisions. Such systems detect the presence of objects ahead and reduce the speed of the vehicle if the driver fails to do so and the vehicle is on a collision course with another vehicle or object. Positioned at the front of the vehicle, distance sensors would detect objects within a certain range and determine if a collision was imminent. The vehicle braking system would then be activated to slow the vehicle. Distance sensing and braking systems will save lives and prevent injuries from front-end MVCs that have been sustained for decades worldwide. 

Automatic braking technologies are already available from several vehicle manufacturers, combining sensors and brake controls to prevent collisions or to reduce speed. Such systems can be considered precursors for fully automated cars that will convey passengers from departure points to preprogramed destinations and assume most if not all of the responsibility for driving. Infrared lasers, microwave radar and video sensors could be deployed on all new cars to reduce speed when necessary to avoid collisions, as well as slow down and space out cars and trucks on freeways, interstate highways and city streets [45]. This would mean that vehicles could travel safely at very high speeds, potentially at hundreds of miles per hour in areas of limited traffic, which would vastly reduce travel times for people and goods and eliminate risks posed by the high-risk or drug-impaired driver—indeed all human operators. Such operating systems could be a boon for the disabled and for those who have to travel great distances, giving all drivers complete freedom to engage in electronic communications and other secondary tasks. Trucking companies would no longer depend on human beings to move goods across the country.

## 6. Driverless Vehicles are in Development

“Smart” safety systems on some current vehicles are already overriding driver operations to prevent collisions. Within a few years, cars are expected to avoid collisions without any type of driver involvement. Public and governmental agencies will expect greater safety features; roads will become more crowded and the average age of drivers will increase. Lightweight and more crashworthy vehicles will be introduced. An eventual goal will be to create the “crashless car”. Indeed, robotic vehicles are already in early production and will be widely available in a few years [45]. These developments will be accomplished in part through computerized systems, glimpses of which we are seeing today through global positioning systems (GPS) and travel planning within vehicles.

Several manufacturing companies and research centers around the world are pioneering these efforts. Google has developed a fleet of Toyota hybrids that are partly capable of driving autonomously. Robotic cars react faster than humans and are not susceptible to distraction or intoxication. Such cars have no blind spots since they can see 360 degrees around them due to a Light Detection and Ranging (LIDAR) system mounted on the roof. The LIDAR system, a rapidly spinning, pulsating laser that detects the constantly changing ranges of all objects around the car to create a three-dimensional map, is coupled to a GPS sensor and a video camera system programed to recognize objects in front of the car such as pedestrians, animals and bicyclists. Three sets of sensors work in concert with Google’s geospatial information database to guide the car and make driving decisions. The self-driving system is activated and deactivated at the slightest touch of the steering wheel or brake. These cars have covered more than 1000 miles without any human control, and 140,000 miles with only occasional human intervention. The only crash occurred when the autonomous car was rear-ended at a stop light [46].

Google’s car uses a set of sensors to detect a “landing strip” or marker on the road when the vehicle is parked; this could be a painted area on the ground or a sign on a wall. Detecting this marker triggers instructions on when and how to activate the self-drive mode of operation. For instance, the self-drive mode could engage when the car enters an interstate highway or disengage when the driver arrives at a destination. After the occupant leaves the vehicle the self-drive mode engages and the car parks itself [47]. One of the primary goals of Google’s self-driving car program is to eliminate the more than 1 million car deaths that occur annually worldwide, virtually all of which are caused by human error. Many technological challenges and issues still need to be resolved, such as operating in the snow, because signals from the road are obscured [48], as well as the implications of driverless cars for future transportation, injury prevention, parking, energy, oil use and climate change [49].

## 7. Policy Implications

In 1942, Hugh DeHaven published his classic paper showing how human beings can survive falls from heights of 50–150 feet [50], which was followed by many technological innovations in vehicle design and transportation systems that enhanced survival in crashes. In the 1930s, physicians Claire L. Straith and C.J. Strickland advocated the use of seat belts and padded dashboards, and Strickland himself founded the Automobile Safety League of America [51]. In 1984, New York was the first state in the U.S. to pass a law requiring seat belt use for passengers in cars. Current estimates are that increased use of seat belts because of such laws saves 10,000 lives per year in the U.S. [52]. Although innovation is rarely accepted without challenge, and litigation has always been central in the struggle to mandate safer cars [53], most vehicle-based reductions in vehicle fatality rates in the U.S. during the last third of the 20th Century were gained by the initial NHTSA safety standards issued from 1968 to 1984 and subsequent voluntary changes in vehicle design and construction by vehicle manufacturers [54].

Policy suggestions for addressing public safety dangers associated with impaired or distracted driving have mainly focused on drivers and driver behavior, e.g., toxicological screening, legal sanctions, mandatory rehabilitation programs, and education efforts [55]. This paper suggests that present and future policy should focus on advances and refinements in technology as a long-term strategy for addressing vehicular and transportation safety, building on the seminal work of Hugh DeHaven, William Haddon and others. However, self-driving vehicle technology has not yet reached the point of being authorized for use by the public for general driving [56].

## 8. Conclusions 

For future dwellers on Earth, even in the next century, it will be a disturbing thought that their forebears in the 20th and 21st centuries drove at high speeds in opposite directions on the same narrow and often hilly roads lacking any type of barrier between vehicles—arrangements that pose high risks for frontal crashes. It is also a matter of concern today that vehicles operated even at low speeds can easily kill, with only the conscious exercise of will power, training and good will to prevent people from killing other drivers and pedestrians. Moreover, few measures are in place to prevent drug or alcohol-impaired drivers from starting their vehicle, and none is available to take over vehicle operations if drivers are suddenly impaired or incapacitated. As Ralph Nader memorably stated nearly 50 years ago in the title of his bestselling book, motor vehicles are “unsafe at any speed” [57], especially, we would add, when operated by human beings. Any type of impairment or distraction—from alcohol and drugs to loss of sleep, fatigue, rage, loss of consciousness or engaging in secondary tasks—can easily lead to a fatal crash. Too much responsibility is currently placed on the driver for ensuring the safety of vehicle operations, vehicle occupants and pedestrians.

Deaths and injuries from motor vehicle accidents remain a significant public health problem. The state of research on injury control in the field of transportation is more advanced compared to that of other categories of injury, and integrated efforts of a mainly technological nature have resulted in dramatic reductions in death rates from MVCs over the past century. However, rather than relying on lowered speed limits and other strategies that require human action to reduce MVCs and injuries, including driver education and regulations to prevent cell phone use and texting, advances in technology can be realistically envisioned that will free human beings from operational responsibility for vehicles. This will further reduce MVCs and injuries on all types of roads and at the same time allow for potentially higher rates of speed than are safe or feasible at present. 
